# The use of miniaturised Bluetooth Low Energy proximity loggers to study contacts among small rodents in agricultural settings

**DOI:** 10.1371/journal.pone.0312553

**Published:** 2025-01-03

**Authors:** Florian Huels, Bram Vanden Broecke, Vincent Sluydts, Lucinda Kirkpatrick, Ivan Herrera Olivares, Hendrik Ennen, Dries Vermeiren, Herwig Leirs, Jens Jacob

**Affiliations:** 1 Julius Kühn-Institut (JKI), Federal Research Centre for Cultivated Plants, Institute for Epidemiology and Pathogen Diagnostics, Rodent Research, Muenster, Germany; 2 Evolutionary Ecology Group (EVECO), University of Antwerp, Wilrijk, Belgium; 3 IoSA Tracking – Internet of Small Animals, Antwerp, Belgium; Southwest University, CHINA

## Abstract

Small rodents can cause problems on farms such as infrastructure damage, crop losses or pathogen transfer. The latter threatens humans and livestock alike. Frequent contacts between wild rodents and livestock favour pathogen transfer and it is therefore important to understand the movement patterns of small mammals in order to develop strategies to prevent damage and health issues. Miniaturised proximity loggers are a newly developed tool for monitoring spatial behaviour of small mammals. The strength of the Bluetooth Low Energy (BLE) signal can be used as an indicator of close contacts between wild rodents and livestock feeding sites, which is relevant for identifying possible transmission routes. This method study focussed on the use of the technology in an agricultural setting as well as dry runs for testing and calibrating this technology in farming environments used for animal husbandry. Results show that the battery life of the loggers was mainly influenced by the pre-set scan interval. Short scan intervals resulted in reduced battery lifespan and should be maximised according to the activity patterns of the target species. Habitat affects BLE signal strength resulting in higher signal strength indoors than outdoors. The height of the location of the loggers positively affected signal strength in livestock stables. Signal reception generally decreased with increasing distance and differed among loggers making calibration necessary. Within habitat specific distances, BLE proximity logging systems can identify contacts among small mammals and between animals and particular structures of interest. These results support the use of BLE based systems in animal husbandry environments and contribute to a body of evidence of validated techniques. In addition, such approaches can provide valuable insights into possible pathogen transmission routes.

## Introduction

Movement and sociality are two fundamental aspects of animal behaviour that have been studied extensively in the past. Spatial movements are essential for animals to forage [1], for mating [2], and to avoid predation [3], while sociality is crucial for their survival and reproduction [4]. Recent studies have shown that these aspects of animal behaviour are closely intertwined, and that movement can have a profound impact on social interactions, and vice versa [5–7]. The most commonly used methods to quantify the movement of wild animals are radio telemetry and GPS-based loggers [8, 9]. Although these systems have many advantages such as detection of long and rapid migrations without visual contact [10], they also have certain limitations, especially in smaller animals such as rodents [11]. For instance, the use of these systems does not allow to quantify movement on a fine spatial scale, and the mass of tracking devices should not exceed 5% of the animal’s body mass to minimize negative behavioural and survival effects [12].

Until recently, information gathering information on contacts between wild animals has therefore been limited to relatively large target species. Additionally, these systems do not allow to measure social behaviour, especially direct social contacts among the individuals. Quantifying the individual’s social behaviour and their position within the underlying social network is therefore essential for gaining a comprehensive understanding of their life history. Pathogen transmission is one of those aspects and social network analysis has been shown to be a powerful tool in the analysis of disease transmission [13].

Indeed, numerous studies have shown that highly connected individuals, which either have significantly more unique contacts than others, or occupy a more central position within the network, are at an increased risk of infection and are pivotal for subsequent disease transmission [13–15]. However, observing the individual’s social behaviours and inferring underlying social networks and the population level is notoriously difficult, especially in small animals and/or those residing in inaccessible environments. The recent and rapid advancements in miniature sensor technology for consumer electronics have led to the development of ultralight proximity loggers, offering new possibilities for studying social interactions among individuals who carry these loggers while simultaneously capturing movement data [16].

This new technology allows users to measure sociality and movement of small mammal species, such as rodents. This is of particular interest in the field of disease ecology, since rodents are known to host a wide variety of pathogens, some of which can be transmitted to livestock and/or humans [17–19]. For example, rodents that were trapped near and in pig and poultry farms in Europe have been found to be infected with a range of different parasites and pathogens such as *Toxoplasma gondii* [20, 21], *Coxiella burnetii* [22], *Salmonella* [23, 24] and *Trichinella* [25]. In Central Europe, there are three species of rodents that are closely associated with humans and livestock: black rat (*Rattus rattus*), brown rat (*Rattus norvegicus*) and house mouse (*Mus musculus*), which pose a threat for both livestock and public health due to the zoonotic diseases they carry [26–29]. Zoonoses comprise a large percentage of all newly identified infectious diseases as well as many existing ones [30], with agricultural and forestry systems constituting a key risk of spill over due to the increased proximity of farm workers to livestock and commensal rodents [31, 32]. A typical example of a widespread zoonotic pathogen carried by commensal rodents is leptospirosis which occurs worldwide [33–35] and affects human health as well as the livestock industry, resulting in significant economic loss [36].

Controlling commensal rodents on farms is challenging and relies heavily on rodenticides, mainly with anticoagulant rodenticides (ARs) that have a delayed mode of action. However, there is a growing concern about the use of these chemicals due to genetic rodenticide resistance [37, 38] and the negative impact on animal welfare [39]. In addition, there is the risk of primary and secondary exposure of non-target species [40–44]. Furthermore, these commensal rodents live in complex, three-dimensional habitats where attractive food sources are plentiful and where suitable places for baiting can be limited [45]. This may result in improper and/or overuse of rodenticides in locations with low rodent activity. Therefore, it is important to identify locations for baiting that are regularly frequented by the target species [46].

The ability to record accurate location data to understand animal movements, behaviour and ecology has steadily increased over the last decades [12]. Telemetry is well established to detect movement patterns of wild animals [47], while different types of logger systems such as GPS-based loggers [12], light loggers [48] or Bluetooth devices [49, 50] have been used in various studies of large wildlife species.

Bluetooth Low Energy (BLE) could be an alternative technique to avoid problems such as a low temporal or spatial resolution, and a high time input in order to gather or analyse data. Miniaturized proximity loggers using Bluetooth signal allow to monitor intra-specific and inter-specific spatial and temporal behaviour of small mammals [11, 51]. Identifying such contacts is essential for understanding the processes of pathogen transfer and for basic behavioural studies of small mammals [50]. Several studies have used Bluetooth logger technology for monitoring and animal movement investigations [16, 49, 51]. However, the use of bespoke BLE proximity loggers in the field is less well documented, including how loggers perform in different environmental conditions. Most prior knowledge is based on theoretical work [51] and aviary trials with birds and on captive *Mastomys natalensis* [11]. Therefore, additional evidence across a range of different habitat types is essential to document the reliability of the technical components for a suitable duration in the field.

This study aimed to test performance and applicability of the ProxLogs BLE proximity logger system (developed by IoSA BV in collaboration with the University of Antwerp) to assess its suitability for detecting coarse spatial movements and contact behaviour in agricultural landscapes dominated by animal husbandry. Dry runs in several habitats were conducted to evaluate effects of manmade obstacles, structural complexity of the habitats, distance between loggers and height of stationary loggers. The results of this study testing how the loggers work in a fairly challenging environment for BLE transmission provide insights into whether they can be used to better understand the movement behaviour of the target species, develop strategies to improve the health of humans, livestock and potentially to optimise baiting strategies.

## Materials and methods

### General functionality

**Fig 1:**
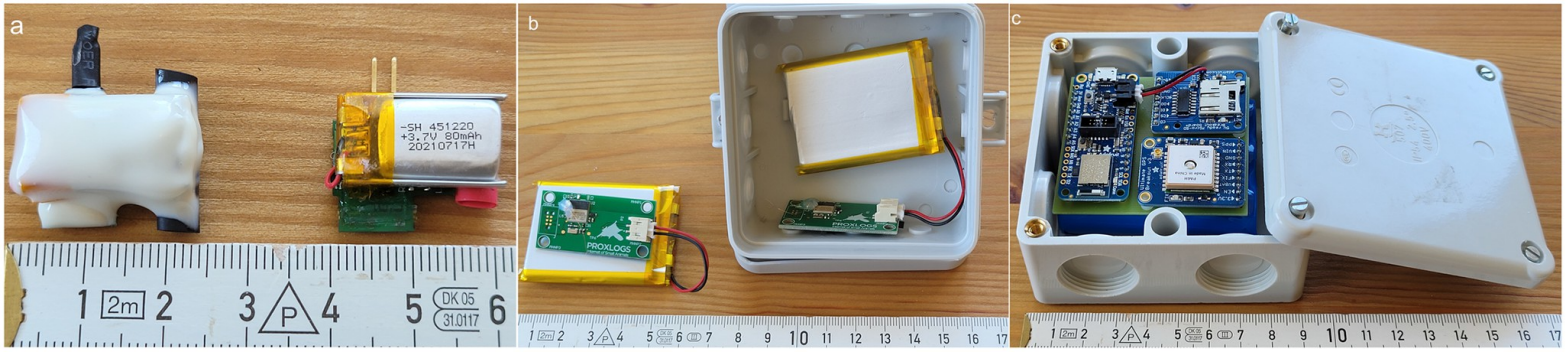
ProxLogs Bluetooth logger system components. Elements of the ProxLogs Bluetooth logger system, (a) mobile logger (rats): as delivered (right), sealed left; (b) stationary logger: as delivered (left), in protective case (right); (c) gateway: in protective hard case.

The BLE ProxLogs logger system consists of mobile loggers to be attached to the animals with cable ties similar to standard radio collars, larger stationary loggers that can be placed at locations of interest in the environment and a gateway device (Fig 1a–1c). The ProxLogs system is designed for signal transmission over short distances, especially when estimating contact between mobile loggers. The loggers alternate between advertising their own identification and scanning their surroundings for other loggers [51]. The scanning rate is set by the user and the advertising interval is calculated based on the scanning rate. The loggers and gateway are controlled through a mobile phone application. If a logger detects another logger in the vicinity, the ID, timestamp and received signal strength indicator (RSSI) of that logger are recorded. Both mobile and stationary loggers broadcast their own ID and scan for other IDs in the environment. Data are stored locally on the loggers and can be downloaded either automatically by a gateway or manually through the mobile phone application. The gateway also updates the logger clock to ensure that loggers are synchronised. In contrast to traditional GPS based systems, the RSSI recorded by BLE loggers gives information about small-scale proximity of animals, and the data are constantly wirelessly transmitted directly to the gateway, so that the loss of the loggers does not result in loss of data. Bluetooth Low Energy signal transmits in waves and can easily be deflected and obstructed by obstacles (metal, water, soil etc.). Due to these multitude of possible sources of bias, loggers have to be carefully calibrated to the particular use case situation.

We sealed all the mobile loggers prior to the trials with a thin layer of epoxy resin (Awapur 2.5, Wagner Dental) for protection. The charging pins were sealed with heat shrink before the sealing process, as they need to be accessible for recharging (Fig 1a). Stationary loggers were equipped with small wire antennas installed by hand. The stationary loggers (8 x 8 x 3.5 cm) and gateways (9 x 9 x 4.5 cm) were placed into waterproof junction boxes to protect them from the elements (Fig 1b and 1c).

### Trials

#### Battery life

Battery life was tested in a climate chamber (Roller, Type HVS 405, installed by Emde-Froend climate technology) at 20°C approximately reflecting mean summer temperature and at 5°C approximately reflecting mean winter temperature in Germany in 2023 [52]. Twenty mobile loggers were used for each temperature regime. Eighteen mobile loggers were scanning either continuously or in hibernation mode without scanning from 9 am to 5 pm (8 hours of inactivity) at a replication of three loggers per scan interval of 10 s, 30 s and 60 s (Table 1). In addition, in each temperature regime, two mobile loggers were advertising only without scanning. For two weeks, the battery status of all loggers was checked daily via the ProxLogs application. The app calculates battery percentage based on the battery voltage. The app then compares that to a comparable curve over percentage. The estimation is a best effort and will not provide a true linear discharge curve.

**Table 1:** Experimental setup climate chamber: Mobile logger settings and their respective scan interval (with hibernation mode (+ H) or without).

Type	n	Temperature [°C]	Scan interval
Rat logger	3	20	10 s
Rat logger	3	20	30 s
Rat logger	3	20	60 s
Rat logger	3	20	10 s + H
Rat logger	3	20	30 s + H
Rat logger	3	20	60 s + H
Rat logger	2	20	Advertising only
Rat logger	3	5	10 s
Rat logger	3	5	30 s
Rat logger	3	5	60 s
Rat logger	3	5	10 s + H
Rat logger	3	5	30 s + H
Rat logger	3	5	60 s + H
Rat logger	2	5	Advertising only

#### Distance/Range detection

The change in BLE signal and the accompanying reliability of the logger system was tested in different target habitats in grassland (open habitat) and in an agricultural equipment shed (closed habitat). Stationary loggers were placed at heights of 1.0 m and 1.6 m on a wooden pole. Two mobile loggers were located on the ground at each distance of 0.5 m, 1.0 m, 1.5 m, 2.0 m, 2.5 m and 3 m away from the pole. The scan interval was 10s to gain as much data points as possible in a short time. This scan interval was applied in all subsequent trials. Data were recorded for 10 min for each of the two heights.

#### Calibration in real conditions

**Fig 2:**
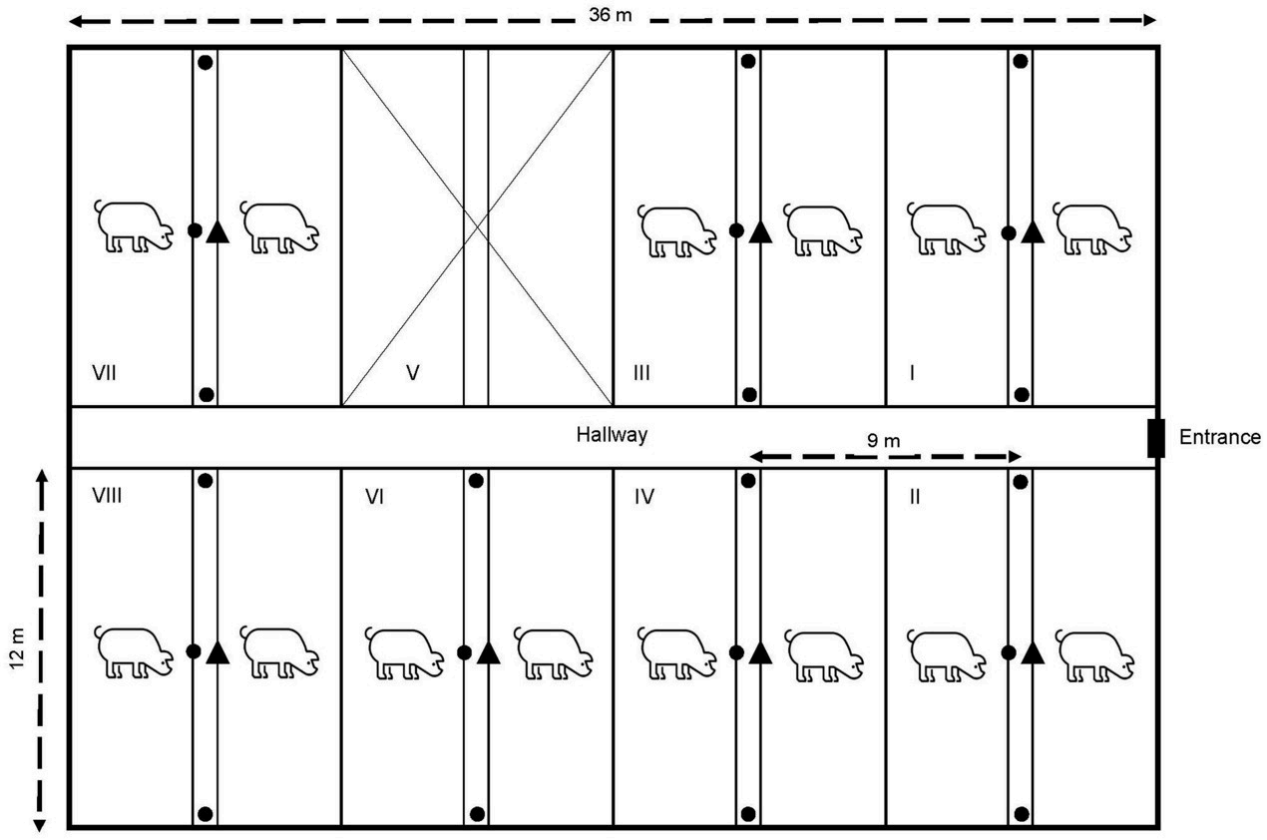
Calibration setup. Experimental setup for the calibration test: placement of one stationary logger (▲) and three mobile loggers (●) in each of the seven occupied stable compartments. The stationary logger in stable IV did not record data for unknown reasons but the mobile loggers in stable IV worked.

The ProxLogs system was calibrated in a mid-size intensive pig farm for breeding and fattening pigs located in Loenhout (17 m a.s.l.), Belgium, in the province of Antwerp from May 08^th^ to 12^th^ 2023. The layout aimed to assess the optimal positioning of loggers to cover a sufficient area with appropriate signal strength. The temperature during the calibration period was between 15 and 20°C and some precipitation on May 9 and 10 (between 7–8 mm, cloud density 80–100%). The farm consisted of four large stable buildings that were used to rear pigs. Besides these stables, there were two additional older buildings. The total size of the farm was 16,000 m^2^ and was surrounded by agricultural landscape used to grow maize and other annual crops typical for the region. The tests were conducted in a typical stable complex for pig husbandry (approximate size of ~ 970 m^2^) (Fig 2). The complex was comprised of eight stables, four on each side. Stable 5 was not available for the trial. A stationary logger was placed at 1 m (medium height) in the corridor in the centre of the stables. Mobile loggers were placed at the front door (distance from SL: 6 m), centre of the corridor (distance from SL: 0 m) and back wall (distance from SL: 6 m) of the stable. Mobile loggers were placed on the ground (1^st^ run), at about 1.0 m in height to cover medium height locations (2^nd^ run) and at about 1.8 m in height to cover high locations (3^rd^ run). Logger data were recorded for 10 min. Distances from stationary loggers to mobile loggers in nearby compartments were also recorded, even if there were physical barriers such as walls or doors in between.

#### Variation among stationary loggers

Given the considerable variation in RSSI and the number of data points collected among the stationary loggers (see below), we wanted to quantify the individual performance of each stationary logger in a controlled environment. A simple test setup similar to the distance/range detection trials was used. Stationary loggers were placed at three heights (0 m, 1.0 m and 1.8 m) on a wooden stand with mobile loggers on the ground at increasing distances (0 m to 2 m in intervals of 0.5 m). Loggers were on scanning mode for 10 min.

### Statistical analysis

The effects of temperature, scan interval and day on battery charge were assessed with a repeated measurement Analysis of Variance (ANOVA). Battery charge was ln-transformed. Loggers were subjects, random effects and nested within scan intervals. Statistically significant effects were kept in the final model and pairwise main effects and interactions tested with a post hoc test (Turkey HSD). For the evaluation of the effect of habitat on signal strength, both outdoors and for calibration in the target habitat, mean RSSI values were multiplied by -1, ln-transformed and a Johnson transformation [53] conducted. The number of data points was also ln-transformed. The effect of outdoor habitat, distance between mobile and stationary loggers and height of stationary loggers were tested with a forward stepwise fully factorial ANOVA. The resulting model contained the statistically significant effects.

In the indoor performance tests, we had a dataset of 14,460 contacts between the mobile loggers and the stationary loggers. We created a linear mixed effects model with the RSSI as the dependent variable with a Gaussian error distribution. The distance between the mobile logger to the stationary logger and the height of the stationary logger were fixed effects, the identity of the mobile and stationary logger were random effects. Repeatability (i.e., proportion of the total variation that can be attributed to variation among the stationary loggers; [54]) was assessed using the rptR package (1,000 parametric bootstraps for interval estimation; version 0.9.22; [54]). We calculated the number of contacts each stationary logger (separately for the different heights) had with the different mobile loggers (no contacts = zero) and fitted a hurdle model using the glmmTMB package (version 1.1.8; [55]). The total number of contacts was the dependent variable and height/distance were the explanatory variables in the presence-absence component. Distance from the mobile logger and the identity of the stationary logger were the explanatory variables in the truncated count component.

Statistical analyses were run with JMP (Version 17.0.0, JMP Statistical Discovery LLC) and R software 4.3.2 [56]. Model assumptions were checked using the DHARMa package (version 0.4.6; [57]). Graphs were created with Excel (Excel 2016, Microsoft Corporation).

## Results

### Battery life

**Fig 3:**
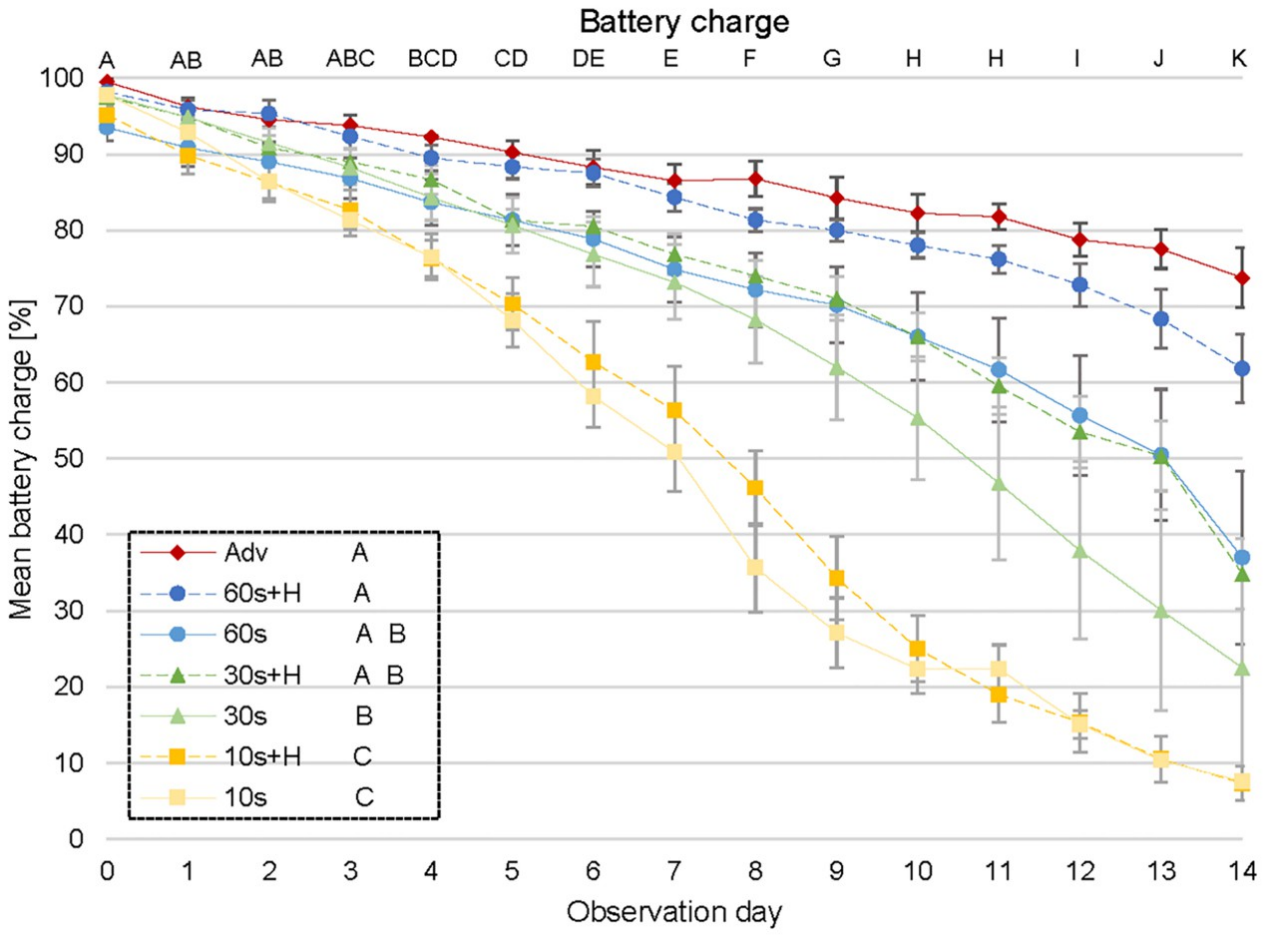
Battery charge. Battery charge of mobile loggers over 14 days for scan intervals 10, 30, 60 s without, with hibernation mode (H), and for a logger advertising only without scanning (Adv). Error bars are standard error. Different capital letters A-K below the figure heading and behind the scan intervals in the legend indicate a statistically significant difference between different letters. Battery charge percentage is calculated by the ProxLogs app based on battery voltage and is an estimation.

Temperature (probability p = 0.46) did not affect battery charge but day (p < 0.001) and scan interval (p < 0.001) did. During week 1, battery charge was largely similar among days but in week 2, this changed mostly due to the decrease in charge for the 10 s scanning mode. Battery charge was lowest for 10 s intervals (>51–58% at end of week 1 and <10% at end of week 2). It was highest for advertising mode only, 60 s interval with and without hibernation mode and 30 s with hibernation mode (75–87% at end of week 1 and 35–74% at end of week 2) and intermediate for 30 s without hibernation mode (>73% at end of week 1 and 23% at end of week 2) (Fig 3). There was no interaction between temperature and day (p = 0.84) but the interaction of scan interval and day mattered (p < 0.001). Here, the pairwise comparison of equal scan intervals with and without hibernation mode revealed that advertising mode significantly differed from 10 s at day 6, from 60 s at day 11 and from 60 s with hibernation mode at day 13. Therefore, advertising mode showed the best battery charge levels for the whole duration of the experiment whereas 10 s was the worst. All others were in between.

#### Distance/Range detection

**Fig 4:**
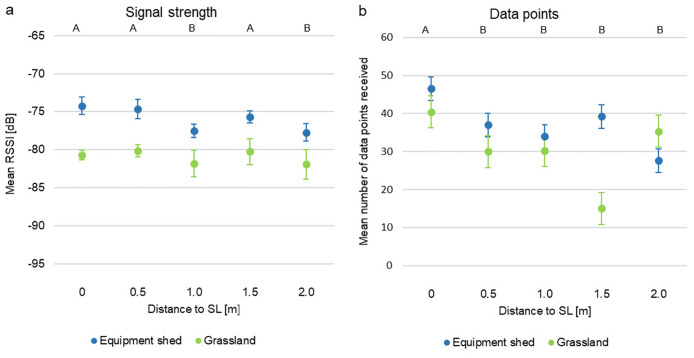
Habitat test. a) Mean RSSI (grey circles) and b) mean number of data points (black circles) per distance in equipment shed and grassland. Error bars are standard error. Different capital letters A-B below the figure heading indicate a statistically significant difference between different letters.

RSSI was affected by habitat (p < 0.001) and distance between mobile logger and stationary logger (p = 0.003), height did not matter (p = 0.522). Mean signal strength was lower in grassland (~ -80 dB) than in the equipment shed (~ -75 dB) (Fig 4a). In grassland, RSSI was higher for distances of 0 m, 0.5 m and 1.5 m (2–3 dB) than for 1.0 m and 2.0 m. In the equipment shed, the trend was similar with higher RSSI values (~2–3 dB) in 0 m, 0.5 m and 1.5 m. There was no effect of habitat (p = 0.551) and height (p = 0.395) on the number of data points received but increasing distance was related to a decreasing number of data points (p = 0.012). In grassland, most data points were recorded at the shortest (0 m) and longest (2.0 m) distances (~40 data points) whereas at the medium distances fewer data points were received especially at 1.5 m (<20 data points) (Fig 4b). In the equipment shed, the closest distance (0 m) yielded the most data points (almost 50). At 2.0 m <30 data points were recorded. There was a strong positive correlation of RSSI and the number of data points in grassland (correlation coefficient rho = 0.540; p < 0.001) and equipment shed (correlation coefficient rho = 0.542; p = 0.001).

#### Calibration in real conditions

**Fig 5:**
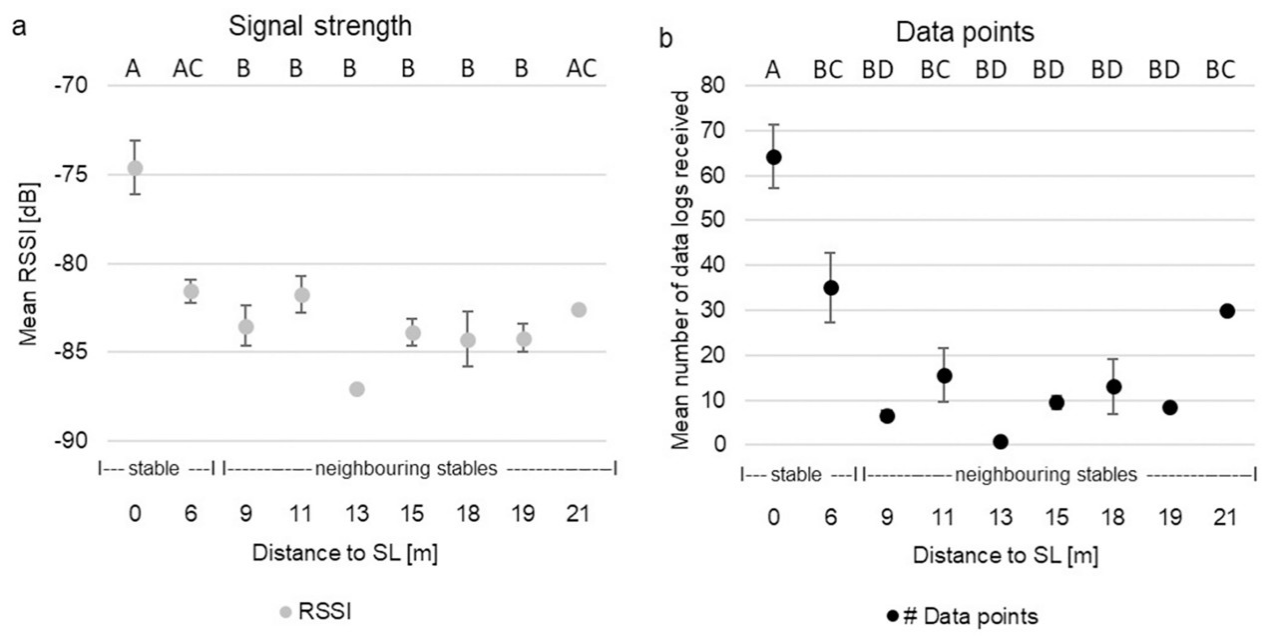
Calibration test. Mean RSSI (a) and mean number of data points (b) over distance. Error bars are standard error. Different letters below the figure heading indicate a significant difference between units.

Whereas height of stationary loggers had no effect on RSSI (p = 0.189) and data points (p = 0.673), the distance between stationary logger and mobile logger affected both RSSI (p = <0.006) and the number of data points (p = <0.007). RSSI was highest at 0 m (>-75 dB) and fluctuated between -82 and -87 dB for all other distances (Fig 5a). The number of data points was higher at 0, 6 and 21 m (30–64 data points) and was lower for all other distances (1–16 data points) (Fig 5b). There was a strong positive correlation between RSSI and the number of data points at medium height (correlation coefficient rho = 0.702; p < 0.001) and high position (correlation coefficient rho = 0.787; p < 0.001) but not at ground level (p = 0.383).

#### Variation among stationary loggers

There were consistent differences among the loggers in RSSI (Repeatability = 41.1%, 95%CI = 14.4–61.2%, p < 0.001). Mean RSSI decreased with distance (estimate ± SE = -1.901 ± 0.671, t = -2.833, p = 0.014) and height (0m-1m: -5.213 ± 0.131, t = -39.785, p < 0.001; 0m-1.8m: -5.550 ± 0.129, t = -42.969, p < 0.001). The probability of having no contact between the stationary and mobile logger decreased when the stationary loggers were elevated (0m-1m: -1.526 ± 0.295, z = -5.180, p < 0.001; 0m-1.8m: -2.074 ± 0.310, z = -6.685, p < 0.001). The distance between the mobile logger and the stationary logger increased the probability of no contact (0.720 ± 0.099, z = 7.302, p < 0.001) and the number of contacts (-0.249 ± 0.054, z = -4.641, p < 0.001). Additionally, there were differences in the number of contacts between the stationary loggers (*χ*^*2*^ = 22.160, p < 0.002).

## Discussion

Bluetooth Low Energy proximity loggers are a new tool that can be used to investigate contacts among animals and to identify small-scale habitat use. However, to ensure that loggers provide accurate data, the reliability of data recording needs to be ascertained in a range of different environments.

We showed that the battery life of mobile loggers depends significantly on the settings and especially the pre-set scan interval confirming the findings of Kirkpatrick et al. [11] and Berkvens et al. [51]. Short scan intervals seem necessary for investigating small mammal species such as rats due to their high movement speed [58] but this will lead to a higher battery discharge [51]. Scan intervals in the range of 30–60 s seemed to balance the need for the collection of an appropriate number of data points in a short time without consuming too much battery charge, and resulted in loggers functioning over a period of at least two weeks with such a scan interval. Compared to other tagging systems of similar size, the BLE logger battery life is longer than equivalent sized GPS tags and shorter than radio tags [59]. Our study confirms that the hibernation mode has an additional positive effect on battery life of mobile loggers and can be useful in fieldwork with species that tend to be inactive at a certain time of the day. Generally, Norway rats are considered to be a nocturnal species [60–62]. However, rats on farms can adapt their individual circadian activity pattern and shift into diurnal activity [27, 63]. For species where the activity phase is not linked to a specific time of day, hibernation mode cannot be applied without running the risk of missing important information. However, for other rodent species such as the largely nocturnal bank vole (*Clethrionomys glareolus*) or the diurnal wood mouse (*Apodemus sylvaticus*) [64], hibernation mode seems a good option to save battery and increase data recording time. Temperatures of 5/20°C did not affect battery life of mobile loggers. However, considerably higher/lower temperatures may yield a different result. Batteries generally discharge more quickly under colder conditions [65], which may matter for the use of the mobile loggers with Norway rats and other commensal rodents that nest in various environments with highly different ambient temperature [28].

ProxLogs was designed to record contacts between individuals using BLE signals. However, as we see in this study, BLE signal is inherently noisy and assigning a consistent and accurate relationship between RSSI and distances is not possible. Instead, distance bins need to be calculated that can be used to coarsely estimate contacts, and these distance bins need to be calibrated across the different habitat types that might be expected to occur across the study system. Synanthropic species like the Norway rat are well adapted to different environmental conditions [26, 28] and will use buildings as well as outdoor environments. Therefore, we tested the ProxLogs system across the different representative habitats that we were likely to encounter in our study system. The current logger system did not work equally in all habitat types and structures but did record data at appropriate distances between mobile and stationary loggers. There were strong differences in individual stationary logger performance within and between environmental conditions, for example, signal strength was consistently weaker in open grassland compared to inside stables. This can be explained by the principles of radio-frequency propagation, reflection, and absorption [66]. In open areas like grasslands, RF-signals spread out and weaken following the free-space path loss (FSPL) model [67]. Vegetation will further increase attenuation of the signal due to absorption, scattering and diffraction [68]. Metal sheds on the other hand, can act as Faraday cages, reflecting RF signals internally. This can create a multipath propagation environment where the signal bounces off walls, ceiling, and floor before reaching the receiver. These reflections can lead to constructive interference at certain points, and/or provide alternative paths when there is an obstruction between two loggers, enhancing the signal strength. This is known as the multipath effect [66]. While this can increase the signal strength with which the signal is received, it can also create more noise in the signal, making accurately determining distance bins more challenging. As seen in Fig 5, distinguishing between 6m and 19m distance in the stables based on RSSI alone would not be possible, therefore, this would need to be accounted for when deciding on where to locate stationary loggers and how to assign distance bins.

The study confirmed previous assumptions (ProxLogs User Manual) concerning the impact of height of the position of the stationary logger on RSSI, particularly inside the stable. Locations between approximately one and two meters above the ground achieved the best results. This can be explained by the wave properties of the BLE signal and the basic physical principles of wave propagation [69] influencing the relative orientation between the logger devices [70], which explains why loggers at elevated positions received data over larger distances and at a higher signal strength than for stationary loggers placed close to the ground. This should be taken into account in future studies that intend to place loggers within buildings. The noisiness of BLE and exponential signal strength loss with distance [71] is an important consideration when using BLE based proximity logging systems in complex structural environments. Ideally, the logger system will cover complete rooms/reasonably large sections of the target building or large outdoor areas without significant blind spots. For the stables used in this study, one stationary logger per stable compartment was sufficient. In other environments such as larger stable compartments or grasslands, more stationary loggers would be necessary to cover the proposed study area.

The reliability of a BLE based proximity logger system is also influenced by the ability of the loggers to successfully transmit and receive signals to/from other mobile loggers [51]. The body of the collared rodent itself can absorb some of the signal and reduce range significantly [72]. Likewise, the ground itself and spatial obstacles in the environment can negatively influence the proliferation of BLE signal because they can cause reflection, diffraction, absorption, and scattering of the radio waves [73]. Over short distances within a building and outdoors, the RSSI can be used to estimate whether a close contact has occurred. However, at larger distances, the extent to which RSSI can be confidently assigned to distances is much more variable and is an inherent property of BLE signal. In studies where the interest is in assessing close contacts between individuals, this may not be an issue and reflects the original purpose for which ProxLogs were designed. In grassland, signals between mobile loggers were received until a distance of about 3 m, while in buildings, the distance as twice as long. Therefore, we consider the distances sufficient when detecting contacts relevant for pathogen transmission. However, we would caution that calibration in study specific habitat types is important to understand how the RSSI may differ in different environmental settings.

Further tests are needed to shed light on the effect of other habitat types that may matter for fieldwork with small rodents such as forests, sewer systems and crop fields. In this study, we also found consistent differences in performance among the stationary loggers, particularly in the RSSI and number of data points received when logging mobile loggers. This necessitated a prior grouping of all loggers by performance. While some variation in performance is always going to occur due to manufacturing tolerances, environmental factors, and the presence of obstacles that can affect signal propagation, it is important to attempt to minimise this as much as possible. Antenna were hand soldered onto stationary loggers for this experiment as there was a manufacturing defect with the original antenna and there were time constraints with the project. Ensuring that future iterations do not require hand soldered antenna but instead use antenna that are soldered at the point of manufacture will decrease the variation in transmission capabilities between the different loggers.

Nevertheless, based on our results, environmental variation (complexity, structure, type, height, etc.) and logger performance variation leads to the suggestion that for the most accurate results, loggers need to be calibrated individually in the field. It is not possible to make a general assumption about the functionality, performance or range of all loggers in different habitat types.

## Conclusion

Proximity logger systems using Bluetooth Low Energy signal can be suitable tools to detect contacts among individuals and between individuals and certain locations. Adjusting scan interval and hibernation mode to optimise battery life increases their utility in different situations. In our study system, the loggers were able to provide data on contacts in a variety of different habitats including inside buildings such as animal stables. Over short distances, mobile and stationary loggers were able to collect many data points but as the distance between the loggers increases the ability of the loggers to accurately record contacts decreases. However, this is unlikely to be important if the focus is on close contacts relevant for pathogen transmission. In our experiment, there was considerable variation among stationary loggers regarding the number of logs and the received signal strength of those logs. Therefore, it was necessary to calibrate each stationary logger individually in advance to optimize performance in the field. Recent use in fieldwork with rodents shows promising performance of the logger system (unpublished data) and further studies could help to optimise the logger system. This technique has the potential to reveal contacts between livestock and small mammals and might contribute to a better understanding of small animal movements and pathogen transmission pathways on farms and in other habitats.

## Supporting information

S1 Data Data from experimental tests (battery charge, habitat test and calibration test). (ZIP)

## Abbreviations

AR: *Anticoagulant rodenticides*: Rodent killing poisons that work by preventing blood from clotting. Results in death days after ingestion of lethal dose.
BLE: *Bluetooth Low Energy*: Low energy-based wireless communication system via Bluetooth.
GPS: *Global Positioning System*: Satellite-based radio navigation system providing geolocation and time information data to specific receiver. Developed by the United States Department of Defence.
RSSI: *Received Signal Strength Indicator*: Estimation of the signal strength received from another device by a radio frequency device [11].
